# Epidemiology and risk factors for pneumonia severity and mortality in Bangladeshi children <5 years of age before 10-valent pneumococcal conjugate vaccine introduction

**DOI:** 10.1186/s12889-016-3897-9

**Published:** 2016-12-07

**Authors:** Shampa Saha, Md Hasan, Lindsay Kim, Jennifer L. Farrar, Belal Hossain, Maksuda Islam, ASM Nawshad Uddin Ahmed, M. Ruhul Amin, Mohammed Hanif, Manzoor Hussain, Shams El-Arifeen, Cynthia G. Whitney, Samir K. Saha

**Affiliations:** 1 Child Health research Foundation, Dhaka Shishu Hospital, Sher-E Bangla Nagar, Dhaka, 1207 Bangladesh; 2 1600 Clifton Road, NE, MS A34, Atlanta, GA 30329 USA; 3 1600 Clifton Road, NE, MS C25, Atlanta, GA 30329 USA; 4 International Centre for Diarrhoeal Disease Research, Bangladesh, Dhaka, 1212 Bangladesh; 5 Department of Microbiology, Bangladesh Institute of Child Health, Dhaka Shishu Hospital, Dhaka, 1207 Bangladesh

**Keywords:** Pneumonia, Epidemiology, Severity, Mortality, *Streptococcus pneumoniae*

## Abstract

**Background:**

Pneumonia is the leading infectious cause of morbidity and mortality in young children in Bangladesh. We present the epidemiology of pneumonia in Bangladeshi children <5 years before 10-valent pneumococcal conjugate vaccine introduction and investigate factors associated with disease severity and mortality.

**Methods:**

Children aged 2–59 months admitted to three Bangladeshi hospitals with pneumonia (i.e., cough or difficulty breathing and age-specific tachypnea without danger signs) or severe pneumonia (i.e., cough or difficulty breathing and ≥1 danger signs) were included. Demographic, clinical, laboratory, and vaccine history data were collected. We assessed associations between characteristics and pneumonia severity and mortality using multivariable logistic regression.

**Results:**

Among 3639 Bangladeshi children with pneumonia, 61% had severe disease, and 2% died. Factors independently associated with severe pneumonia included ages 2–5 months (adjusted odds ratio [aOR] 1.60 [95% CI: 1.26–2.01]) and 6–11 months (aOR 1.31 [1.10–1.56]) relative to 12–59 months, low weight for age (aOR 1.22 [1.04–1.42]), unsafe drinking water source (aOR 2.00 [1.50–2.69]), higher paternal education (aOR 1.34 [1.15–1.57]), higher maternal education (aOR 0.74 [0.64–0.87]), and being fully vaccinated for age with pentavalent vaccination (aOR 0.64 [0.51–0.82]). Increased risk of pneumonia mortality was associated with age <12 months, low weight for age, unsafe drinking water source, lower paternal education, disease severity, and having ≥1 co-morbid condition.

**Conclusions:**

Modifiable factors for severe pneumonia and mortality included low weight for age and access to safe drinking water. Improving vaccination status could decrease disease severity.

## Background

Pneumonia is the leading cause of death from an infectious cause in children <5 years, with the majority of deaths occurring in developing countries, in part, due to limited access to healthcare and public health interventions [1, 2]. Over ten million new cases of pneumonia in children <5 years are diagnosed annually in Bangladesh [3]. The pneumococcal (PCV) and the *Haemophilus influenzae type b* (Hib) conjugate vaccines effectively reduce the burden of pneumonia by targeting two of the most common bacterial etiologies [4]. However, while Hib vaccine has been widely introduced, PCV has yet to be introduced in many countries in the Southeast Asia Region, [5] even though the World Health Organization (WHO) recommends PCV inclusion in childhood national immunization programs [6].

In Bangladesh, Hib vaccine was introduced in 2009 with significant reductions of both pneumonia and meningitis [7, 8]. The Bangladesh Expanded Programme on Immunization introduced 10-valent PCV (PCV10) on a 6, 10, and 18 week schedule in March 2015, becoming the second country in the region (after Pakistan) to do so. The objective of this analysis is to present the epidemiology of pneumonia in Bangladeshi children <5 years prior to PCV10 introduction and investigate risk factors associated with disease severity and mortality. This analysis will also serve as baseline data for the future documentation of PCV10 impact on pneumonia in this population and to evaluate the potential for other prevention options.

## Methods

### Study setting

The study was conducted using the WHO-supported Invasive Bacterial Disease (IBD) surveillance network in three hospitals, a system established in 2004 [9, 10]. Dhaka Shishu (Children’s) Hospital (DSH) and Shishu (Children) Shasthya Foundation Hospital are the two largest pediatric hospitals in Bangladesh; they are located in urban Dhaka and have 600 and 200 pediatric beds, respectively. DSH and Shishu (Children) Shasthya Foundation Hospital provide treatment for 47 and 5% of admitted patients at no cost, respectively. Kumudini Women’s Medical College Hospital is located 65 km north of Dhaka in the rural sub-district of Mirzapur and has 60 pediatric beds. Kumudini Women’s Medical College Hospital provides medical care at subsidized costs to all patients. All three hospitals provide primary care; DSH also serves as the tertiary care center for pediatrics in the country. The hospitals have similar standards of care for pneumonia patients. Chest X-rays are done for the majority of the pneumonia cases. Non-severe cases are treated with first-line antibiotics (e.g., amoxicillin and gentamicin), and severe cases are treated with second-line antibiotics (e.g., ceftriaxone) according to WHO [11]. Severe pneumonia cases are provided with supplemental oxygen.

### Case recruitment, enrollment, study definitions, and data collection

All children aged 2–59 months who were admitted to the study hospitals were interviewed and screened for specific signs and symptoms of pneumonia, meningitis, and sepsis by trained study physicians. Children admitted to another hospital for >24 h prior to admission at a study hospital were excluded from hospital surveillance. Based on clinical criteria, admitted children were categorized as having pneumonia, severe pneumonia, meningitis, or sepsis according to WHO recommended IBD surveillance case definition [12]. These definitions were not mutually exclusive.

A child was enrolled in IBD surveillance if they met the case definition for pneumonia, severe pneumonia, meningitis, or sepsis and if specimens (blood or cerebrospinal fluid) were collected [13]. Specimens were collected at the treating physician’s discretion, according to routine clinical practice [13]. A chest X-ray was obtained per clinical discretion of the treating physician and interpreted by a staff radiologist or pediatrician. Since chest X-rays were not interpreted in a standardized way, radiological information was not analyzed or reported.

For this analysis, only hospitalized cases with pneumonia and severe pneumonia and a corresponding specimen were included. Cases who were diagnosed as lab-confirmed meningitis (etiology detected by any laboratory method) or probable bacterial meningitis (>9 leukocyte count on cerebrospinal fluid cytology) were excluded from the analysis. Demographic, clinical, laboratory, and vaccine history data were collected using a standardized questionnaire. Weight of the children was obtained on admission using calibrated scales after removal of any heavy clothing. Vaccination status of children eligible for pentavalent vaccines (combined *Haemophilus influenzae* type b, hepatitis B, diphtheria, pertussis, and tetanus vaccine) was obtained from parents by verbal report. Children were considered age-eligible for pentavalent vaccine if they were 6–14 weeks old during 2009 and onwards (i.e., period that pentavalent vaccine was available). If a child was age-eligible for vaccine and was reported to have received vaccine doses by the parent, then the child was considered “up-to-date for age” using the child’s reported age to determine how many doses the child should have received. Children with pneumonia/severe pneumonia, but without a corresponding clinical specimen, were excluded in this analysis because they did not have minimal data collected (i.e., basic demographics, clinical information, and risk factors). In order to obtain information about final diagnosis and outcome, all pneumonia cases (regardless of collection of clinical specimen) were followed up until discharge or death.

Two definitions were used to describe pneumonia severity. “Pneumonia” was defined as history of coughing or difficulty breathing and age-specific tachypnea (≥50 and ≥40 breaths/min for 2–11 month-olds and 12–59 month-olds, respectively) without any of the following signs: inability to drink or breastfeed, vomiting with a final discharge diagnosis code for pneumonia or severe pneumonia, convulsions, prostration/lethargy, chest indrawing, or stridor in a calm child [12]. “Severe pneumonia” was defined as history of coughing or difficulty breathing and ≥1 of the aforementioned signs. Because the sign, “vomiting everything” was not specifically captured in the abstraction form, we included children with a history of vomiting who also had a final hospitalization diagnosis of pneumonia or severe pneumonia to improve specificity for this sign. “Clinical pneumonia” was used to refer to both “Pneumonia” and “Severe pneumonia”.

### Laboratory methods

Blood specimens were cultured at the respective sentinel hospital laboratories as described elsewhere [14]. Pneumococcal isolates were identified using standard methods and preserved in media containing skim milk, tryptone, glucose, and glycerin at −70 °C [14]. All isolates detected in site laboratories were sent to the Microbiology Department of DSH where identification was confirmed. Pneumococcal isolates were serotyped by Quellung method as described previously [15].

### Data analysis

Data from January 2011 to December 2013 were included in this analysis. Characteristics of clinical pneumonia cases were compared by severity and mortality. Weight for age was calculated using the WHO Child Growth Standards, which utilized z-scores stratified by gender to define weight for age categories [16]. Differences in proportions were contrasted using Pearson’s chi-square test or Fisher’s exact test, as appropriate. Bivariate odds ratios and corresponding 95% confidence intervals were calculated to quantify the association between possible risk factors with pneumonia severity and mortality. Statistically significant factors, defined as a *p-*value < 0.05, were retained for multivariable logistic regression analysis with pneumonia severity and mortality as outcomes. Backward stepwise logistic regression with *p* < 0.05 was performed to obtain the final models. Data analysis was conducted using STATA 13.1 (StataCorp LP, College Station, TX, USA).

## Results

During January 2011–December 2013, an estimated 66 139 children aged 2–59 months were admitted to the surveillance hospitals. Fourteen percent (*n* = 8979) met the clinical definition of pneumonia or severe pneumonia; of these, 3840 (43%) had a specimen collected during their hospitalization and were enrolled in the study. Among these 3840 children, 201 were excluded as they were diagnosed as either laboratory confirmed or probable bacterial meningitis, and the remaining 3639 were included in this analysis.

Major reasons for non-enrollment in IBD surveillance included: physician did not advise blood culture (*n* = 4954/5139, 96%); specimen collection failure (*n* = 67, 1%) and being discharged, leaving against medical advice, death, or being referred to another hospital before a study physician had the opportunity to enroll the child (*n* = 39, 0.8%); 79 (1.5%) cases were not enrolled for other reasons (e.g., refusal to participate or data missing). Children that did not have a specimen collected differed from children who had a specimen collected. Children who did not have specimens collected were younger (8.5 vs. 12.3 months), had more co-morbid conditions (7.9% vs. 5.7%), and experienced longer hospitalizations (5.5 vs. 4.8 days) with more deaths (2.7% vs. 1.7).

**Fig. 1:**
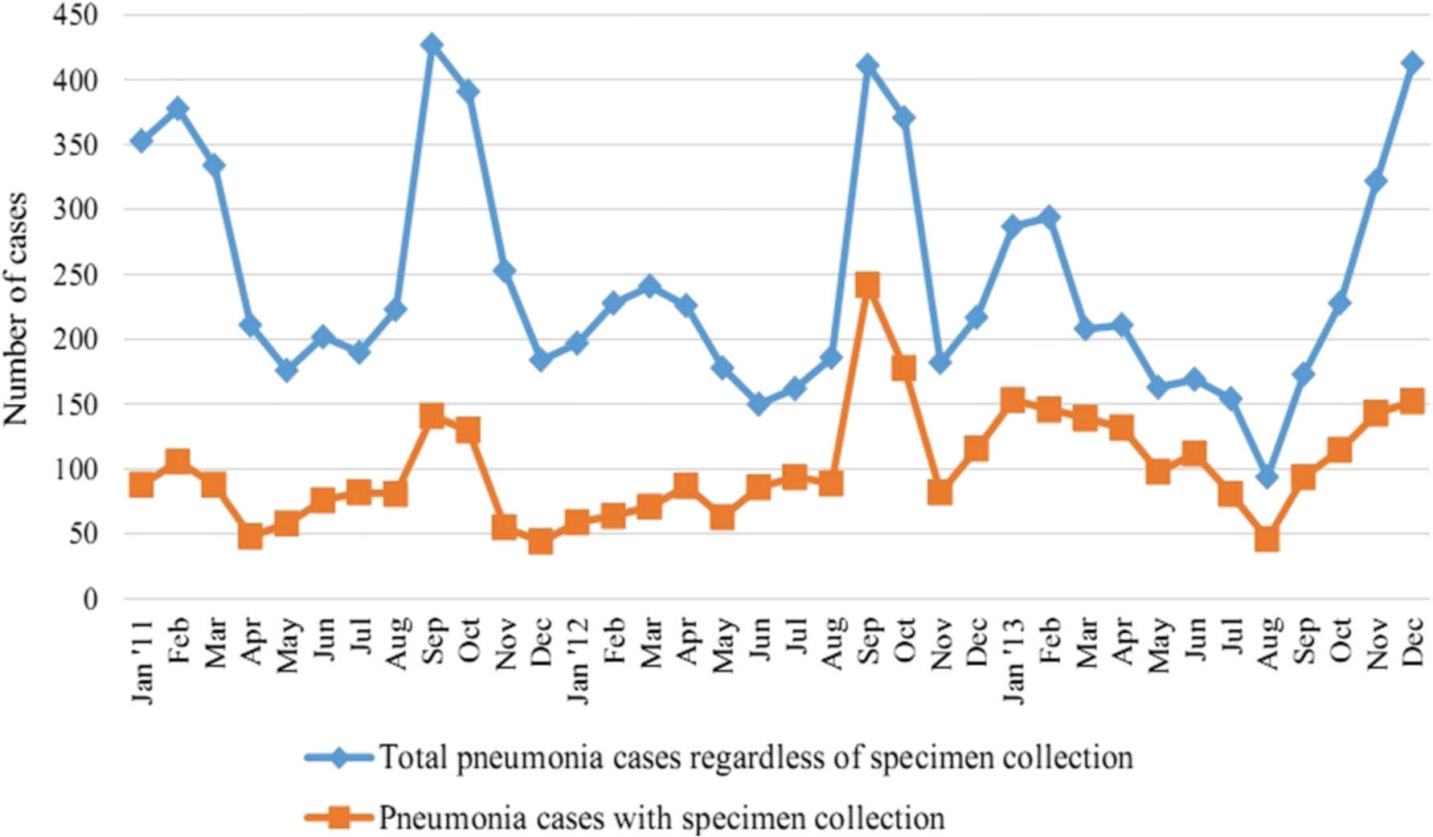
Total number of pneumonia cases with or without specimen collection by month, January 2011-December 2013. This figure shows the total number of pneumonia cases with or without specimen collection by month during January 2011 through December 2013 at the surveillance hospitals. There appears to be some seasonality with cases peaking during the August, September, and October months

We observed seasonal variation in clinical pneumonia admissions to the surveillance hospitals (Fig. 1) and a similar variation in the number of cases enrolled in IBD surveillance (i.e., children with clinical pneumonia and a specimen). The highest numbers of clinical pneumonia cases occurred during the late monsoon and autumn season (3 months, August–October), though the peak in 2013 continued through December.

### Characteristics of pneumonia cases

According to the WHO IBD surveillance case definition, 1409 (39%) and 2230 (61%) of children had pneumonia and severe pneumonia, respectively (Table 1). Sixty percent (*n* = 2168/3639) of children with clinical pneumonia were aged 2–11 months, and 36% were female. Thirty-one percent of children with clinical pneumonia were low weight for age. Children who had severe pneumonia were more frequently younger in age (*p* < 0.001), had lower weight for age (*p* = 0.02), reported an unsafe drinking water source (*p* < 0.001), had more access to hygienic latrines (*p* = 0.03), and had fathers with higher educational levels (*p* = 0.001) and mothers with lower educational levels (*p* < 0.001) compared to children with pneumonia (Table 1).

**Table 1:** Demographic and clinical characteristics of children with clinical pneumonia, by severity

Characteristic	Total *N* = 3639	Pneumonia^a^ *N* = 1409 n (%)	Severe pneumonia^a^ *N* = 2230 n (%)	*P*-value for comparison of pneumonia vs. severe pneumonia
Female gender	1291 (35.5)	475 (33.7)	816 (36.6)	0.08
Age				<0.001
2–5 months	1169 (32.1)	348 (24.7)	821 (36.8)	
6–11 months	999 (27.5)	401 (28.5)	598 (26.8)	
12–59 months	1471 (40.4)	660 (46.8)	811 (36.4)	
Weight for age				0.02
Normal weight for age	2515 (69.1)	1005 (71.3)	1510 (67.7)	
Low weight for age	1120 (30.8)	402 (28.5)	718 (32.2)	
Unknown	4 (0.1)	2 (0.1)	2 (0.1)	
Number of family members in household				0.39
2–4	1580 (43.4)	596 (42.3)	984 (44.1)	
≥ 5	2051 (56.4)	811 (57.6)	1240 (55.6)	
Unknown	8 (0.2)	2 (0.1)	6 (0.3)	
Drinking source^b^				<0.001
Safe water	3340 (91.8)	1333 (94.6)	2007 (90.0)	
Unsafe water	296 (8.1)	76 (5.4)	220 (9.9)	
Unknown	3 (0.1)	0 (0)	3 (0.1)	
Latrine type^c^				0.03
Hygienic	2111 (58.0)	785 (55.7)	1326 (59.5)	
Unhygienic	1525 (41.9)	624 (44.3)	901 (40.4)	
Unknown	3 (0.1)	0 (0)	3 (0.1)	
Father’s education^d^				0.001
None	450 (12.4)	173 (12.3)	277 (12.4)	
Some primary	171 (4.7)	80 (5.7)	91 (4.1)	
Some secondary	1611 (44.3)	666 (47.3)	945 (42.4)	
Higher than secondary	1401 (38.5)	489 (34.7)	912 (40.9)	
Unknown	6 (0.2)	1 (0.1)	5 (0.2)	
Mother’s education^d^				<0.001
None	300 (8.2)	105 (7.5)	195 (8.7)	
Some primary	211 (5.8)	84 (6.0)	127 (5.7)	
Some secondary	2206 (60.6)	918 (65.2)	1288 (57.8)	
Higher than secondary	916 (25.2)	300 (21.3)	616 (27.6)	
Unknown	6 (0.2)	2 (0.1)	4 (0.2)	
Supplemental oxygen use during hospitalization	2184 (60.0)	753 (53.4)	1431 (64.1)	<0.001
Blood specimens collected prior to antibiotic administration	1254 (34.5)	444 (31.5)	810 (36.3)	0.003
Number of co-morbid conditions^e^				
0	3433 (94.3)	1349 (95.7)	2084 (93.5)	0.004
1+	206 (5.7)	60 (4.3)	146 (6.5)	
Pentavalent vaccination status per parent report (*N* = 3,433; 1331 pneumonia, 2102 severe pneumonia) ^f^				<0.001
None	198 (5.8)	51 (3.8)	147 (7.0)	
Partially vaccinated for age	741 (21.6)	209 (15.7)	532 (25.1)	
Fully vaccinated for age	2451 (71.4)	1056 (79.3)	1395 (66.4)	
Unknown	43 (1.3)	15 (1.1)	28 (1.3)	
Outcome				<0.001
Discharged	3401 (93.5)	1342 (95.2)	2059 (92.3)	
Referred	41 (1.1)	11 (0.8)	30 (1.3)	
Left against medical advice	127 (3.5)	39 (2.8)	88 (3.9)	
Died	63 (1.7)	11 (0.8)	52 (2.3)	
Unknown	7 (0.2)	6 (0.4)	1 (0)	

Percentages may not sum to 100% due to rounding

^a^Case definitions: 1) Pneumonia: history of coughing or difficulty breathing and age-specific tachypnea without any of the following signs, including inability to drink or breastfeed, vomiting with a final discharge diagnosis code for pneumonia or severe pneumonia, convulsions, prostration/lethargy, chest indrawing, or stridor in a calm child); 2) Severe pneumonia: history of coughing or difficulty breathing and ≥1 of the aforementioned signs. Because the sign, “vomiting everything” was not specifically captured in the abstraction form, we included children with a history of vomiting who also had a final hospitalization diagnosis of pneumonia or severe pneumonia to improve specificity for this sign

^b^Safe water sources include piped water (boiled) and tube well or deep tube well. Unsafe water sources include piped water (not boiled), surface water (e.g., pond, tank, lake, river, streams), and other sources not specified

^c^Hygienic latrines include septic tank/modern toilet and pit latrine (water sealed). Unhygienic latrines include pit latrine (not sealed), open or hanging latrine, and bush or field

^d^Education categories were defined as the following: some primary (1–4 years), some secondary (5–9 years), higher than secondary (10 years or more)

^e^Co-morbid conditions: congenital heart disease, thalassemia, nephrotic syndrome, Down’s syndrome, protein energy malnutrition (PEM) and tuberculosis

^f^Vaccination status for pentavalent vaccines was collected from parents by verbal report. To be included, the child had to be age-eligible for the vaccine

Cough (*n* = 3482, 96%) was most frequently reported by caregivers of children with clinical pneumonia followed by fever (*n* = 2702, 74%) and difficulty breathing (*n* = 1595, 44%). Chest indrawing (*n* = 1159, 52%) and convulsions (38%, *n* = 841) were frequently found among children with severe pneumonia. Among all pneumonia cases, 2184 (60%) were given supplemental oxygen during hospitalization; the requirement of supplemental oxygen increased with increasing disease severity (53% for pneumonia and 64% for severe pneumonia, *p* < 0.001). Thirty-five percent (*n* = 1254) of children received antibiotics prior to blood collection. Children with severe pneumonia had higher frequencies of reporting ≥1 co-morbid conditions (p0.004). Ninety-three percent (*n* = 3192) of 3433 age-eligible children were reported as being partially (*n* = 741, 22%) or fully (*n* = 2451, 71%) vaccinated with pentavalent vaccine.

Several factors were significantly associated with pneumonia severity (Table 2). On multivariable analysis, factors independently associated with severe pneumonia included younger age, low weight for age, unsafe drinking water source, and having a father with higher than secondary education. Having a mother with some secondary education and receiving all doses of pentavalent vaccine were protective against severe pneumonia (Table 2).

**Table 2:** Factors associated with severe pneumonia rather than pneumonia^a^: results of univariable and multivariable analysis

	Unadjusted odds ratio (OR)	95% confidence interval (CI)	Adjusted OR	95% CI
Age				
2–5 months	1.91	1.63–2.26	1.60	1.26–2.01
6–11 months	1.21	1.04–1.43	1.31	1.10–1.56
12–59 months	ref	--	ref	--
Low weight for age	1.19	1.03–1.37	1.22	1.04–1.42
Unsafe water	1.92	1.46–2.51	2.00	1.50–2.69
No hygienic latrine	0.85	0.77–0.98	--	--
Father’s education^b^				
None	ref	--	ref	--
Some primary	0.69	0.48–0.98	--	--
Some secondary	0.88	0.71–1.08	--	--
Higher than secondary	1.14	0.92–1.42	1.34	1.15–1.57
Mother’s education^b^				
None	ref	--	ref	--
Some primary	0.81	0.57–1.17	--	--
Some secondary	0.76	0.59–0.97	0.74	0.64–0.87
Higher than secondary	1.11	0.84–1.45	--	--
Pentavalent vaccination status per parent’s report^c^				
None	ref	--	ref	--
Partially vaccinated for age	0.88	0.62–1.26	--	--
Fully vaccinated for age	0.46	0.33–0.64	0.64	0.51–0.82
Number of co-morbid conditions^d^				
0	ref	--	ref	--
1+	1.58	1.16–2.14	--	--

^a^Case definitions: 1) Pneumonia: history of coughing or difficulty breathing and age-specific tachypnea without any of the following signs, including inability to drink or breastfeed, vomiting with a final discharge diagnosis code for pneumonia or severe pneumonia, convulsions, prostration/lethargy, chest indrawing, or stridor in a calm child); 2) Severe pneumonia: history of coughing or difficulty breathing and ≥1 of the aforementioned signs. Because the sign, “vomiting everything” was not specifically captured in the abstraction form, we included children with a history of vomiting who also had a final hospitalization diagnosis of pneumonia or severe pneumonia to improve specificity for this sign

^b^Education categories were defined as the following: some primary (1–4 years), some secondary (5–9 years), higher than secondary (10 years or more)

^c^Vaccination status for pentavalent vaccines was collected from parents by verbal report. To be included, the child had to be age-eligible for the vaccine

^d^Co-morbid conditions: congenital heart disease, thalassemia, nephrotic syndrome, Down’s syndrome, protein energy malnutrition (PEM) and tuberculosis

### Pneumonia mortality

Illness outcome was recorded in 3632 (99%) cases; among these, 63 (2%) resulted in death. Younger age, lower weight for age, and several other factors were associated with mortality on univariable analysis (Table 3). Factors independently associated with mortality among children with clinical pneumonia on multivariable analysis included age <11 months, low weight for age, unsafe drinking water source, disease severity, and presence of at least one co-morbid condition (Table 4). Higher than secondary education of the father was protective against pneumonia mortality (Table 4).

**Table 3:** Demographic and clinical characteristics of children with clinical pneumonia, by outcome (*N* = 3,632)

Characteristic	Total *N* = 3632 n (%)	Died *N* = 63 n (%)	Survived *N* = 3569 n (%)	*P*-value
Female gender	1289 (35.5)	29 (46.0)	1260 (35.3)	0.08
Age				<0.001
2–5 months	1168 (32.2)	34 (54.0)	1134 (31.8)	
6–11 months	998 (27.5)	17 (27.0)	981 (27.5)	
12–59 months	1466 (40.4)	12 (19.0)	1454 (40.7)	
Weight for age				<0.001
Normal weight for age	2511 (69.1)	17 (27.0)	2494 (69.9)	
Low weight for age	1117 (30.8)	46 (73.0)	1071 (30.0)	
Unknown	4 (0.1)	0 (0)	4 (0.1)	
Number of family members in household				<0.001
2–4	1578 (43.4)	23 (36.5)	1555 (43.6)	
≥ 5	2046 (56.3)	38 (60.3)	2008 (56.3)	
Unknown	8 (0.2)	2 (3.2)	6 (0.2)	
Drinking source^a^				<0.001
Safe water	3334 (91.8)	51 (81.0)	3283 (92.0)	
Unsafe water	295 (8.1)	11 (17.5)	284 (8.0)	
Unknown	3 (0.1)	1 (1.6)	2 (0.1)	
Latrine type^b^				<0.001
Hygienic	2107 (58.0)	29 (46.0)	2078 (58.2)	
Not hygienic	1522 (41.9)	33 (52.4)	1489 (41.7)	
Unknown	3 (0.1)	1 (1.6)	2 (0.1)	
Father’s education^c^				<0.001
None	450 (12.4)	16 (25.4)	434 (12.2)	
Some primary	170 (4.7)	4 (6.3)	166 (4.7)	
Some secondary	1605 (44.2)	30 (47.6)	1575 (44.1)	
Higher than secondary	1401 (38.6)	12 (19.0)	1389 (38.9)	
Unknown	6 (0.2)	1 (1.6)	5 (0.1)	
Mother’s education^c^				<0.001
None	300 (8.3)	9 (14.3)	291 (8.2)	
Some primary	211 (5.8)	8 (12.7)	203 (5.7)	
Some secondary	2199 (60.5)	37 (58.7)	2162 (60.6)	
Higher than secondary	916 (25.2)	7 (11.1)	909 (25.5)	
Unknown	6 (0.2)	2 (3.2)	4 (0.1)	
Supplemental oxygen use during hospitalization	2180 (60.0)	60 (95.2)	2120 (59.4)	<0.001
Severity^d^				<0.001
Mild pneumonia	1403 (38.6)	11 (17.5)	1392 (39.0)	
Severe pneumonia	2229 (61.4)	52 (82.5)	2177 (61.0)	
Number of co-morbid conditions^e^				<0.001
0	3426 (94.3)	46 (73.0)	3380 (94.7)	
1+	206 (5.7)	17 (27.0)	189 (5.3)	
Pentavalent vaccination status up-to-date for age^f^ (*N* = 3,429; 58 dead, 3371 alive)				0.001
None	198 (5.5)	9 (15.5)	189 (5.6)	
Partially vaccinated	740 (20.6)	20 (34.5)	720 (21.4)	
Fully vaccinated	2448 (68.3)	27 (46.6)	2,421 (71.8)	
Unknown	43 (1.2)	2 (3.6)	41 (1.2)	

Percentages may not sum to 100% due to rounding

^a^Safe water sources include piped water (boiled) and tube well or deep rube well. Unsafe water sources include piped water (not boiled), surface water (e.g., pond, tank, lake, river, streams), and other sources not specified

^b^Hygienic latrines include septic tank/modern toilet and pit latrine (water sealed). Unhygienic latrines include pit latrine (not sealed), open or hanging latrine, and bush or field

^c^Education categories were defined as the following: some primary (1–4 years), some secondary (5–9 years), some higher than secondary (10 years or more)

^d^Severity definitions: 1) Pneumonia: history of coughing or difficulty breathing and age-specific tachypnea without any of the following signs, including inability to drink or breastfeed, vomiting with a final discharge diagnosis code for pneumonia or severe pneumonia, convulsions, prostration/lethargy, chest indrawing, or stridor in a calm child); 2) Severe pneumonia: history of coughing or difficulty breathing and ≥1 of the aforementioned signs. Because the sign, “vomiting everything” was not specifically captured in the abstraction form, we included children with a history of vomiting who also had a final hospitalization diagnosis of pneumonia or severe pneumonia to improve specificity for this sign

^e^Co-morbid conditions: congenital heart disease, thalassemia, nephrotic syndrome, Down’s syndrome, protein energy malnutrition (PEM) and tuberculosis

^f^Vaccination status for pentavalent vaccines was collected from parents by verbal report. To be included, the child had to be age-eligible for the vaccine

**Table 4:** Factors associated with mortality among children with clinical pneumonia: results of univariable and multivariable analysis

	Unadjusted odds ratio (OR)	95% confidence interval (CI)	Adjusted OR	95% CI
Age				
2–5 months	3.63	1.87–7.05	3.52	1.51-8.18
6–11 months	2.10	1.00–4.41	2.58	1.04-6.41
12–59 months	ref	--	ref	--
Low weight for age	6.30	3.59–11.04	4.77	2.48–9.16
Number of family members in household				
2–4	ref	--	ref	--
≥ 5	1.28	0.75–2.16	--	--
Unsafe water^a^	2.49	1.29–4.83	2.17	1.02–4.62
Non-hygienic latrine	1.59	0.96–2.62	--	--
Father’s education^b^				
None	ref	--	ref	--
Some primary	0.65	0.22–1.98	--	--
Some secondary	0.52	0.28–0.97	--	--
Some higher than secondary	0.23	0.11–0.49	0.49	0.24–0.97
Mother’s education^b^				
None	ref	--	ref	--
Some primary	1.27	0.48–3.36	--	--
Some secondary	0.55	0.26–1.15	--	--
Higher than secondary	0.25	0.09–0.60	--	--
Severity^c^				
Mild pneumonia	ref	--	ref	--
Severe pneumonia	3.02	1.57–5.81	2.35	1.12–4.92
Number of co-morbid conditions^d^				
0	ref	--	ref	--
1+	6.61	3.72–11.75	3.19	1.68–6.05
Pentavalent vaccination status up-to-date for age^e^				
None	ref	--	ref	--
Partially vaccinated	0.58	0.26–1.30	--	--
Fully vaccinated	0.23	0.11–0.51	--	--

^a^Safe water sources include piped water (boiled) and tube well or deep rube well. Unsafe water sources include piped water (not boiled), surface water (e.g., pond, tank, lake, river, streams), and other sources not specified

^b^Education categories were defined as the following: some primary (1–4 years), some secondary (5–9 years), some higher than secondary (10 years or more)

^c^Severity definitions: 1) Pneumonia: history of coughing or difficulty breathing and age-specific tachypnea without any of the following signs, including inability to drink or breastfeed, vomiting with a final discharge diagnosis code for pneumonia or severe pneumonia, convulsions, prostration/lethargy, chest indrawing, or stridor in a calm child); 2) Severe pneumonia: history of coughing or difficulty breathing and ≥1 of the aforementioned signs. Because the sign, “vomiting everything” was not specifically captured in the abstraction form, we included children with a history of vomiting who also had a final hospitalization diagnosis of pneumonia or severe pneumonia to improve specificity for this sign

^d^Co-morbid conditions: congenital heart disease, thalassemia, nephrotic syndrome, Down’s syndrome, protein energy malnutrition (PEM) and tuberculosis

^e^Vaccination status for pentavalent vaccines was collected from parents by verbal report. To be included, the child had to be age-eligible for the vaccine

### Laboratory results

Sixty-two (2%) blood cultures were positive for a pathogen among children with clinical pneumonia (Table 5). The proportion positive varied by surveillance site, with DSH recovering significantly more pathogens from blood culture (isolation rate 4%) than Shishu (Children) Shasthya Foundation Hospital (isolation rate 3%) and Kumudini Women’s Medical College Hospital (isolation rate 0.8%). The most common organisms isolated were *Streptococcus pneumoniae* (*n* = 21, 34%), *Salmonella typhi* (*n* = 13, 21%), and *Klebsiella pneumoniae* (*n* = 6, 10%) (Table 5). There were 2 (3%) *Haemophilus influenzae* isolates, and both were nontypeable. The most common pneumococcal serotypes isolated among children with pneumonia were 23 F (*n* = 3, 14%), 6B (*n* = 2, 10%), 14 (*n* = 2, 10%), 19 F (*n* = 2, 10%), and 8 (*n* = 2, 10%) (Table 6). Serotypes included in PCV10 were detected in 67% (*n* = 14) of all blood culture positive pneumococcal pneumonia cases (Table 6). No serotypes included in 13-valent PCV, but not in PCV10 (i.e., serotypes 3, 6A, and 19A), were identified among blood cultures.

**Table 5:** Blood culture results, by pneumonia severity

	Pneumonia^a^ *N* = 1402 n (%)	Severe pneumonia^a^ *N* = 1947 n (%)	Total *N* = 3349 n (%)
No growth	1307 (93)	1794 (92)	3101 (93)
Contamination^b^	76 (5)	110 (8)	186 (7)
Total positive blood cultures	19 (1)	43 (2)	62 (2)
*S. pneumoniae*	9 (47)	12 (28)	21 (34)
*S. typhi*	3 (16)	10 (23)	13 (21)
*K. pneumoniae*	2 (11)	4 (9)	6 (10)
*Acinetobacter sp.*	0 (0)	4 (9)	4 (6)
*S. aureus*	2 (11)	2 (5)	4 (6)
*Enterobacter sp.*	0 (0)	4 (9)	4 (6)
*Non-typeable H. influenzae*	1 (5)	1 (2)	2 (3)
*S. paratyphi-A*	1 (5)	2 (5)	3 (5)
*Pseudomonas sp.*	0 (0)	2 (5)	2 (3)
*Salmonella sp.*	1 (5)	0 (0)	1 (2)
*E.coli*	0 (0)	1 (2)	1 (2)
*Streptococcus sp.*	0 (0)	1 (2)	1 (2)

Percentages may not sum to 100% due to rounding

^a^Case definitions: 1) Pneumonia: history of coughing or difficulty breathing and age-specific tachypnea without any of the following signs, including inability to drink or breastfeed, vomiting with a final discharge diagnosis code for pneumonia or severe pneumonia, convulsions, prostration/lethargy, chest indrawing, or stridor in a calm child); 2) Severe pneumonia: history of coughing or difficulty breathing and ≥1 of the aforementioned signs. Because the sign, “vomiting everything” was not specifically captured in the abstraction form, we included children with a history of vomiting who also had a final hospitalization diagnosis of pneumonia or severe pneumonia to improve specificity for this sign

^b^The following isolates were considered as contaminants: *Streptococci viridans, Micrococcus* sp., *Bacillus* sp., diphtheroids, coagulase-negative staphylococci, and *Candida* sp.^30^

**Table 6:** Serotypes of *S. pneumoniae* isolates from pneumonia cases (*N* = 21)

Serotype or group	N (%)
PCV10-type	14 (67)
1	1 (5)
4	1 (5)
5	1 (5)
6B	2 (10)
7 F	1 (5)
9 V	0 (0)
14	2 (10)
18C	1 (5)
19 F	2 (10)
23 F	3 (14)
Additional PCV13-type	0 (0)
3	0 (0)
6A	0 (0)
19A	0 (0)
Serogoup 6, undifferentiated	1 (5)
Non-vaccine serotypes	6 (29)
2	1 (5)
8	2 (10)
12A	1 (5)
15A	1 (5)
33 F	1 (5)

## Discussion

This study reported a large series of clinical pneumonia among children <5 years in Bangladeshi hospitals before PCV introduction. Among children with specimen collected and culture data available, approximately 61% had severe pneumonia; however, the case-fatality rate was low (2%). Factors independently associated with both pneumonia severity and mortality included young age, low weight for age, an unsafe drinking water source, and paternal education.

Most of our findings are consistent with risk factor analyses in previous pneumonia studies [17, 18]. Similarly, risk factors associated with mortality, such as low weight for age, lack of access to safe drinking water, and presence of at least one co-morbid condition, were also consistent with previous studies [19, 20]. Not surprisingly, increased mortality of children <5 years was positively related to severity of pneumonia as seen in other studies [19, 21]. Interestingly, we found no significant association between pentavalent vaccination status and pneumonia mortality on multivariable modeling; however, pentavalent vaccination status was found to be protective for severe pneumonia. The low number of pneumonia deaths might have made finding an association between vaccination and mortality difficult. It is also possible that since vaccine history was obtained by parental verbal report, misclassification of vaccination status may have made it difficult to find an association.

Modifiable factors for both severity and mortality included weight for age (a marker of nutritional status) and access to safe drinking water. Improving vaccination status could decrease disease severity. There is potential to prevent severe pneumonia and poor outcomes by targeting interventions for these aforementioned factors. For example, nutritional status can be improved by promoting optimal breastfeeding practices with adequate complementary feeding, encouraging micronutrient supplementation and reducing the incidence of low birth weight by improving maternal nutrition, thereby, reducing pneumonia severity and mortality risk [22]. The integrated Global Action Plan for the Prevention and Control of Pneumonia and Diarrhoea also recommends improving breast feeding practice, vaccination coverage, access to safe drinking water, sanitation, hygiene practice, and household air quality in order to prevent pneumonia deaths [23].

However, one factor that we cannot fully explain is the discrepancy between the association of paternal education with pneumonia severity and mortality. For severity, higher paternal education was associated with increased odds of severe pneumonia, while higher paternal education was linked to decreased odds for mortality. This could be a result of unmeasured confounding, where educational level represents some other unmeasured factor. Another potential explanation might be that highly educated fathers might be too busy to take the child to the hospital, leading to more severe disease with late diagnosis and treatment; conversely, the educated father might be able to afford better healthcare, therefore, leading to better outcomes and less mortality. Additionally, the exclusion of more severe and younger cases from enrollment might also have contributed to this discrepant finding. In terms of maternal education, previous literature has linked higher maternal education levels to better health outcomes, including reduced childhood mortality [24].

Our blood culture data suggest that, after the introduction of Hib conjugate vaccine in Bangladesh, Hib is no longer a major cause of blood culture confirmed pneumonia, and pneumococcus is the leading cause. Unpublished lab data of the same study sites also indicate near-elimination of invasive Hib diseases after introduction of Hib conjugate vaccine. Globally, a meta-analysis of several randomized clinical trials showed reductions in both clinical and radiologically-confirmed pneumonia after PCV introduction [25]. Among children <5 years, 9-valent and 7-valent PCV reduced clinical pneumonia by 7% in the Gambia and by 4% in the United States, respectively [26, 27]. While these reductions seem relatively small, they might still have large impact given the burden of pneumonia globally, and specifically, in resource poor countries [28]. Additionally, reductions in community-acquired radiologically-confirmed pneumonia were also seen in South Africa, [29] the Gambia, [26] and the United States [27]. Data from observational studies conducted post-PCV introduction showed significant reductions in pneumonia hospitalizations. An additional reduction may be seen in those not targeted to receive the vaccine due to indirect effects after the PCV immunization program matures in Bangladesh, similar to what has been documented in the United States [30].

This study has several limitations. First, our study was not designed to estimate the population-based burden of pneumonia, only the number and type of cases treated in the three hospitals included in this analysis. Second, we included only pneumonia cases with culture for analysis due to limited risk factor data on pneumonia cases without culture. By doing so, we might have selected less severe cases and biased our results as we did find differences in severity between children with and without cultures as previously discussed. The nonenrollment of severe cases might underestimate our mortality findings and weaken the associations of risk factors for pneumonia. Third, blood cultures were collected in less than half of pneumonia patients, and the low recovery rate of microorganisms from blood culture might underestimate the true burden of invasive pneumococcal pneumonia. Low recovery rates might have occurred due to relatively high antibiotic use prior to blood culture collection or other factors such as difficulty collecting adequate blood volume. Another study from Bangladesh also reported similar low recovery rates [13]. Clinical trials of PCV in Africa have been shown to work as “probe studies”, illustrating the large fraction of pneumonia caused by pneumococcus that is not culture-positive [26, 29]. Similar to these studies, a large fraction of pneumococcal disease in Bangladesh are not likely to be detected by culture. Fourth, data on vaccination were collected from verbal report of the parents, which might be subjected to recall bias and misclassification of vaccination status. However, during the study period, Bangladesh did not have pneumococcal conjugate vaccine in its national immunization program and national coverage for Hib conjugate vaccine was >90%, which led to near elimination of Hib diseases [8, 31].

## Conclusions

Our study demonstrated a large number of clinical pneumonia episodes, with no lab-confirmed Hib pneumonia, among young children in Bangladesh. Interventions that focus on improving nutritional status and access to safe water might decrease pneumonia severity and mortality among young children. In addition, our analysis found that PCV10 could potentially prevent 67% of documented pneumococcal pneumonia cases based on blood culture results. Improved diagnostic tools could also play a major role in determining etiology of childhood pneumonia and developing preventive strategies. Continued surveillance is crucial to monitor the trend of pneumonia with the improvement of nutritional status, supply of safe water, and the introduction of PCV in Bangladesh, as well as to assess the impact of newly-introduced PCV.

## Abbreviations

aOR: Adjusted odds ratio
DSH: Dhaka Shishu Hospital
Hib: *Haemophilus influenzae type b*
IBD: Invasive bacterial disease
PCV: Pneumococcal conjugate vaccine
PCV10: 10-valent pneumococcal conjugate vaccine
WHO: World Health Organization
